# Does Sodium Knowledge Affect Dietary Choices and Health Behaviors? Results From a Survey of Los Angeles County Residents

**DOI:** 10.5888/pcd14.170117

**Published:** 2017-11-22

**Authors:** George Dewey, Ranjana N. Wickramasekaran, Tony Kuo, Brenda Robles

**Affiliations:** 1 Division of Chronic Disease and Injury Prevention, Los Angeles County Department of Public Health, Los Angeles, California; 2 Department of Epidemiology, University of California, Los Angeles, Fielding School of Public Health, Los Angeles, California; 3 Department of Family Medicine, David Geffen School of Medicine at UCLA, Los Angeles, California; 4 Department of Community Health Sciences, UCLA Fielding School of Public Health, Los Angeles, California

## Abstract

**Introduction:**

In 2010, the Los Angeles County Department of Public Health launched a local sodium-reduction initiative to address the rising prevalence of high blood pressure (hypertension) and related cardiovascular conditions in the population. To inform this effort, we evaluated self-reported knowledge and health behaviors related to sodium intake among Los Angeles County residents.

**Methods:**

We administered 3 cross-sectional Internet panel surveys on knowledge about dietary sodium to a sample of Los Angeles County adults, at intervals from December 2014 through August 2016. Multinomial and logistic regression models were constructed to describe associations between sodium knowledge and self-reported health behaviors.

**Results:**

A total of 7,067 panel subjects clicked into the online survey, and 2,862 completed the survey (adjusted response rate = 40.5%). Only 102 respondents (3.6%) were able to accurately report the recommended milligrams of sodium that an average adult should consume daily (1,500 mg to 2300 mg). Knowing about daily sodium intake recommendations was associated with increased odds of using Nutrition Facts labels to make food purchase decisions (adjusted odds ratio [AOR], 3.48; 95% confidence interval [CI], 1.59–7.60) and with decreased odds of taking measures to prevent hypertension (AOR, 0.38; 95% CI, 0.19–0.74).

**Conclusions:**

Los Angeles County residents had a limited knowledge of recommended daily sodium intake. Efforts to increase understanding of these recommendations may encourage wider engagement in healthy behaviors. Health agencies should integrate sodium reduction messages in their diet and nutrition educational efforts.

## Introduction

High blood pressure (hypertension) is the main risk factor for heart disease and stroke, 2 of the leading causes of death among US adults (1). Obesity and diets high in sodium contribute to blood pressure elevation (2). The* Dietary Guidelines for Americans 2015–2020* recommends that healthy adults consume no more than 2,300 mg of sodium per day and that at-risk adults (eg, people with hypertension, people aged 51 years or older, black people) consume less than 1,500 mg of sodium per day (3). The high levels of sodium contained in processed foods, which constitute most food purchases in the United States, can make it difficult for adults to meet these recommendations (4).

Los Angeles County (LAC), the most populous county in the United States, has a high prevalence of chronic diseases associated with excess sodium consumption (eg, 29.3% of LAC adult residents have hypertension) (5). Sodium knowledge in the population is also problematic. A previous assessment showed that less than 10% of LAC adults knew recommendations for daily sodium consumption (6). To date, few studies have examined the relationships between sodium knowledge and health behaviors, and even less is known about these relationships in a large urban population. We evaluated self-reported knowledge and health behavior related to sodium intake among LAC residents to inform an initiative to address the rising prevalence of high blood pressure and related cardiovascular conditions in this population.

## Methods

### Study design

Our study built on a previous assessment of sodium knowledge, attitudes, and behaviors among LAC residents (6) that consisted of a series of 3 cross-sectional Internet panel surveys administered by Global Strategies Group, from December 2014 through August 2016, for the LAC Department of Public Health (DPH). Each survey comprised from 55 to 62 questions distributed across 5 categories: food selection and consumption, support for policy changes related to food environments, nutrition knowledge and awareness, health status, and demographics. Wherever possible, the surveys’ questions were derived from validated questionnaires such as the Behavioral Risk Factor Surveillance System (BRFSS) (7) and the National Health and Nutrition Examination Survey (NHANES) (8). Other questions not addressed by NHANES or BRFSS, such as those pertaining to attitudes toward sodium in foods served in the workplace or at restaurants, were developed internally by DPH.

Because data collection from Internet panels is a continuous process, data from the 3 surveys were combined into one data set. Each survey’s questions were pretested with a pilot group of either 50 or 100 participants to determine accuracy and quality of the survey’s programming. No changes were made after piloting each survey before distribution. Surveys of pilot group participants were included in the final tally of completed surveys. To ensure comparability across surveys, only questions that remained consistent over time were used in the analysis.

### Participants

Participants were LAC residents aged 18 years or older who were able to complete the surveys in either English or Spanish. To ensure a representative sample of county residents, quotas and weights generated by using demographic and geographic data from the 2013 American Community Survey (9) and the 2011 Los Angeles County Health Survey were applied (10). These quotas took into account age, race, sex, income, and LAC Service Planning Area (11). After quota criteria were established, the 3 surveys were distributed to participants by Global Strategy Group. The resulting data were weighted to account for potential undercoverage from the survey’s web-based format and for differential nonresponse resulting from low response rates for certain hard-to-reach demographic groups, such as young residents (particularly those aged 18 to 24 years) and people without computer access. Incentives provided to participants who completed the survey included various gift cards, points programs, or partner products and services at the discretion of the panel provider working with Global Strategies.

Sociodemographic information was collected for all survey participants. Age was converted from a numeric response into a categorical variable in 6 age categories (18–24 y, 25–34 y, 35–44 y, 45–54 y, 55–64 y, and ≥65 y). Race/ethnicity responses were collapsed into 5 categories: African American/black, white, Asian, Hispanic/Latino, and other. Participants were asked to provide their education and annual income level. Body mass index (BMI, measured as weight in kg/height in m^2^) was self-reported. BMI values that were implausible by guidelines of the Centers for Disease Control and Prevention (CDC) (ie, weight >600 lbs or height >8 ft) were excluded from the study (12).

### Measures

Frequencies and weighted percentages were generated for all categorical variables encompassing the demographic characteristics of the survey population, knowledge of nutritional concepts, and health behaviors. To assess participants’ knowledge of daily sodium intake recommendations, participants were asked “How many milligrams of sodium should an average adult consume on a daily basis?” Participants were also asked to identify the number of calories an average adult should consume daily, to compare their knowledge of recommendations for calorie and sodium consumption. To examine their understanding of the consequences of excess sodium consumption, participants were asked, “How harmful do you think consuming salt is for your health?” These questions were used in a previous survey analysis to assess sodium knowledge among LAC residents (6).

General health status was assessed by asking participants how they felt about their general health and whether a doctor or other health care provider had told them to watch their salt intake or told them they had hypertension. Sodium consumption was assessed by asking whether participants added salt to their food and whether they were watching their salt intake. Food purchasing behavior and decision making were assessed by asking how often participants changed their mind about a food purchase on the basis of its sodium content and how often they used Nutrition Facts labels or other food labels during a food purchase. Use of these questions has been described elsewhere (6).

### Analyses

Frequencies and weighted percentages were generated for all categorical variables, which encompassed the population’s demographic characteristics, weight status, knowledge about nutrition, and health behaviors. Logistic and multinomial multiple regressions were constructed to assess associations between knowledge about sodium and health behavior variables. Each regression model controlled for demographic characteristics and included one main predictor per outcome. We used logistic regression to analyze relationships between outcome variables with 2 response levels and used multinomial regressions for outcomes with more than 2 response levels. All analyses were performed using SAS version 9.4 (SAS Institute, Inc). All study protocols and instruments were approved by the LAC DPH institutional review board.

The second and third surveys used 2 sets of questions to assess participants’ ability to correctly read Nutrition Facts labels and to identify high-sodium food items. These questions were not asked in the first survey. Two variables were created by using these sets of questions. The first variable was based on a set of questions that assessed participants’ ability to compare the sodium content of foods from Nutrition Facts labels. The second variable was based on a set of questions that asked participants to identify high-salt foods from a list. For the first variable, 2 groups of Nutrition Facts labels were shown to participants: 1) 2 labels where participants were asked to identify the healthier of 2 food items and 2) 3 labels where participants were asked to identify the item with the least sodium per cup (Figure). Responses to these 2 questions were collapsed into a single variable with 3 values: 1) answering neither question correctly 2) answering one question but not the other correctly, or 3) answering both questions correctly. For the second variable, 10 to 15 foods were shown to participants who were then asked whether the foods contained high, medium, or low amounts of sodium. Only the foods previously promoted as high sodium to LAC residents during the Salt Shocker health marketing campaign (https://www.choosehealthla.com/eat/salt/) were analyzed (13). These included bread, ketchup, cottage cheese, and canned vegetables. For each of these foods, the response “high sodium” was classified as answered correctly, and the responses “medium sodium” or “low sodium” were classified as answered incorrectly. Variables for the 4 items were then collapsed into a dichotomous variable where participants who correctly identified at least 2 of the 4 items were sorted into one category, “gave the correct response,” and participants who correctly identified either one or none of the items were sorted into another, “gave the incorrect response.”

**Figure:**
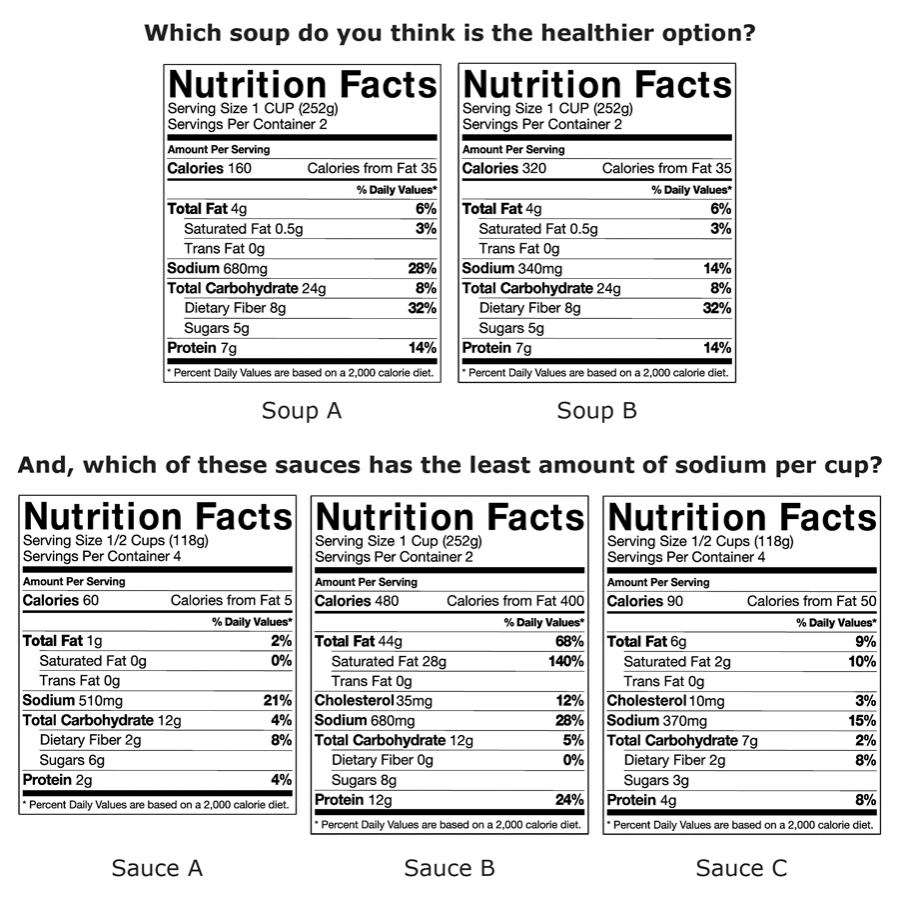
Nutrition Facts labels presented to participants for evaluation, Los Angeles County, Internet panel survey, 2014–2016. Participants were asked to use the 2 labels at the top to select the healthier of the 2 soups, A or B. They were also asked to identify which of the 3 Nutrition Facts labels on the bottom, A, B, or C, had the least sodium per cup.

## Results

Throughout the sampling period, 7,067 panel subjects clicked into the online survey. Of these, 2,862 completed the survey, resulting in an adjusted response rate for all 3 surveys of approximately 40.5%. This adjusted response rate was calculated by dividing the number of completed surveys by the number of eligible participants. Participants were excluded from the final sample if they were younger than 18 years, did not live in LAC, or because of quota criteria.

Participants were evenly distributed across age groups with the largest group aged 25 to 34 years (20.9%). Most were white (40.2%), female (51.3%), had some college or an associate’s degree or bachelor’s degree (54.6%), and had an annual income of $25,000 to $49,999 (22.6%). More than half reported perceiving themselves as overweight (53.5%); similarly, BMI calculations (based on participants’ self-reported heights and weights) showed that 58.7% of participants were overweight or obese (Table 1).

**Table 1:** Demographic Characteristics of Participants (N = 2,862), Internet Panel Survey, Los Angeles County, 2014–2016

Characteristic	No. ( %)^a^
**Sex**	
Male	1,395 (48.7)
Female	1,467 (51.3)
**Age, y**	
15–24	405 (14.2)
25–34	597 (20.9)
35–44	525 (18.3)
45–54	526 (18.4)
55–64	390 (13.6)
≥65	418 (14.6)
**Race/ethnicity**	
African American/black	263 (9.2)
White	1,151 (40.2)
Asian	407 (14.2)
Hispanic/Latino	958 (33.5)
Other	83 (2.9)
**Annual income, $**	
<15,000	294 (10.3)
15,000–24,999	360 (12.6)
25,000–49,999	647 (22.6)
50,000–74,999	488 (17.1)
75,000–99,999	322 (11.3)
100,000–149,999	396 (13.8)
>150,000	300 (10.5)
**Education**	
Less than high school diploma	110 (3.9)
High school diploma or general equivalency diploma	839 (29.5)
Some college	734 (25.8)
Associate’s degree or bachelor’s degree	819 (28.8)
Master’s, doctorate, or other professional degree	344 (12.1)
**Weight, self-reported**	
Underweight	92 (3.2)
Overweight	1,531 (53.5)
The right weight	1,122 (39.2)
Don’t know	117 (4.1)
**Weight, measured, body mass index (kg/m^2^)^b^**	
Underweight	65 (2.4)
Normal	1,062 (38.9)
Overweight or obese	1,603 (58.7)

a Number of participants is unweighted. Percentages are weighted to account for variability in sampling and differential nonresponse. Percentages may not total 100% because of rounding.

b Body mass index was calculated by using the Centers for Disease Control and Prevention’s formula for adults: weight (kg)/height (m^2^) and classified as follows: underweight, <18.5; normal, 18.5–24.9; overweight, 25.0–29.9; obese, >30.0. Implausible weights and heights (ie, weight >600 lbs or height >8 ft) were excluded from analysis (12).

Only 3.6% of participants were able to accurately report the daily recommended sodium intake for an adult (1,500 mg to 2,300 mg). Conversely, 31.7% of participants knew the correct daily calorie intake recommendation. About half (50.7%) believed that consuming salt was somewhat harmful to their health. More than half (54.8%) reported currently watching or reducing their salt intake. Less than a third (31.4%) indicated they had ever been told by a doctor or other health professional to watch their salt intake (Table 2). Although most participants were able to correctly answer 1 of 2 questions regarding Nutrition Facts labels (57.8%), only 21.4% were able to correctly identify at least half of the high-sodium foods presented in the survey. 

**Table 2:** Nutritional Knowledge and Associated Health Behaviors of Participants (N = 2,862), Internet Panel Survey, Los Angeles County, 2014–2016 (N = 2,862)

Question and Answer	No. (%)^a^
**Health Behaviors**	
**In general, how would you rate your health?**	
Excellent/very good	421 (14.7)
Good	1,215 (42.5)
Fair/poor	1,226 (42.8)
**Has a doctor or other health professional ever told you to watch your salt intake?**	
Yes	900 (31.4)
No	1,962 (68.6)
**Are you doing anything to control or prevent high blood pressure?**	
Yes	746 (26.1)
No	2,116 (73.9)
**Are you currently watching or reducing your salt intake?**	
Yes	1,567 (54.8)
No	1,293 (45.2)
**How often do you add salt to your food?**	
Never/rarely	1,465 (51.2)
Sometimes	874 (30.5)
Always/most of the time	523 (18.3)
**How often do you change your mind about buying a food product because of its salt content?**	
Never/rarely	881 (32.6)
Sometimes	715 (26.5)
Always/most of the time	1,107 (41.0)
**How often do you use a food label or Nutrition Facts label to help you decide what food to purchase?**	
Never/rarely	892 (31.2)
Sometimes	805 (28.1)
Always/most of the time	1,165 (40.7)
**Nutritional Knowledge**	
**What impact, if any, do you think consuming salt has on your health?**	
Not harmful	555 (19.4)
Somewhat harmful	1,449 (50.7)
Harmful	858 (30.0)
**How many calories should an average adult consume on a daily basis?**	
Between 1,800 and 2,400 (acceptable range)	914 (31.7)
Answers outside acceptable range	1,948 (68.1)
**How many milligrams of sodium should an average adult consume on a daily basis?**	
Between 1500 mg and 2300 mg (acceptable range)	102 (3.6)
Answers outside acceptable range	2,760 (96.5)

a Number of participants is unweighted. Percentages are weighted to account for variability in sampling and differential nonresponse. Percentages may not total 100% because of rounding.

Participants who believed that consuming salt was very harmful to their health compared with those who believed sodium consumption was only somewhat harmful had increased odds of not adding salt to their food (adjusted odds ratio [AOR], 2.91; 95% confidence interval [CI], 2.16–3.92) and changing one’s mind about a food purchase based on its salt content (AOR, 2.30; 95% CI, 1.70–3.10) (Table 3). Participants who believed consuming salt was very harmful to their health compared with those who believed it was only somewhat harmful had increased odds of watching or reducing their salt intake (AOR, 2.71; 95% CI, 2.09–3.49) and decreased odds of doing anything to control or prevent high blood pressure (AOR, 0.84; 95% CI, 0.64–1.07). Conversely, participants who did not believe consuming salt was harmful to their health compared with those who believed it is somewhat harmful were found to have lower odds of changing their mind about purchasing a food item because of its sodium content (AOR, 0.47; 95% CI, 0.33–0.67). The odds of taking measures to prevent high blood pressure (ie, exercising regularly, controlling or trying to lose weight, reducing sodium intake, taking medicine prescribed by a doctor, or avoiding alcohol or cigarettes) among participants who accurately reported the daily recommended sodium intake for adults was lower than for those who could not accurately report the recommendation (AOR, 0.38; 95% CI, 0.19–0.74). Participants who accurately reported the recommended daily sodium intake had higher odds of reporting watching or reducing salt intake (AOR, 1.59; 95% CI, 0.87–2.89). These participants also had higher odds of having had a doctor or health professional recommend watching salt intake (AOR, 1.24; 95% CI, 0.68–2.24). In addition, knowing about daily sodium intake recommendations was associated with increased odds of using Nutrition Facts labels to make food purchase decisions (AOR, 3.48; 95% CI, 1.59–7.60). In subanalyses, participants who were able to accurately identify high-sodium foods when shown Nutrition Facts labels or a panel of 4 high-sodium foods showed increased odds of changing their mind about buying foods because of their sodium content (Nutrition Facts questions, AOR, 2.35; 95% CI, 1.60–3.45, 4-food panel, AOR, 1.47; 95% CI, 1.01–2.12) (Table 3). Similarly, these same participants had increased odds of currently watching or reducing their salt intake (Nutrition Facts label questions AOR, 1.49; 95% CI, 1.04–2.13; 4-food panel, AOR, 2.14; 95% CI, 1.42–3.48).

**Table 3:** Multinomial Regression Analysis^a^ of Participant (N = 2,862) Responses, Internet Panel Survey, Los Angeles County, 2014–2016

Independent Variable (Answer)	Dependent Variables (Responses)							
	Self-Reported Health Status^b^		How often do you add salt to your food?^c^		How often do you change your mind about buying a food product because of its salt content?^d^		How often do you use a food label or Nutrition Facts label to help you decide what food to purchase?^d^	
	Excellent/Very Good	Fair/Poor	Never/Rarely	Always/Most of the Time	Sometimes	Always/Most of the Time	Sometimes	Always/Most of the Time
**Main Analysis — All 3 Surveys**								
**What impact, if any, do you think consuming salt has on your health?^e^**								
Very harmful	1.13 (0.78–1.64)	1.10 (0.84–1.43)	2.91 (2.16–3.92)	1.27 (0.85–1.91)	1.36 (0.96–1.91)	2.30 (1.70–3.10)	0.83 (0.58–1.17)	1.63 (1.20–2.23)
Not harmful	1.03 (0.66–1.60)	1.25 (0.92–1.68)	0.99 (0.72–1.38)	1.22 (0.82–1.79)	0.47 (0.33–0.67)	0.49 (0.35–0.69)	0.61 (0.43–0.87)	0.82 (0.58–1.16)
**How many calories should an average adult consume on a daily basis?**								
Answered within acceptable range (1,800–2,400)	0.85 (0.60–1.20)	1.15 (0.90–1.47)	1.03 (0.80–1.33)	0.80 (0.58–1.11)	1.11 (0.83–1.47)	1.03 (0.80–1.33)	0.98 (0.72–1.32)	1.41 (1.06–1.86)
**How many milligrams of sodium should an average adult consume on a daily basis?**								
Answered within acceptable range (1,500–2,300)	0.89 (0.28–2.85)	1.12 (0.61–2.05)	0.68 (0.36–1.27)	0.40 (0.17–0.96)	1.87 (0.86–4.06)	1.72 (0.89–3.32)	1.61 (0.67–3.87)	3.48 (1.59–7.60)
**Subanalysis — Surveys 2 and 3 (N = 2,014)**								
**High sodium food panel**								
Correctly identified at least 50% of items (ie, panel of 4 high-sodium foods) as high sodium	1.62 (1.00–2.61)	1.10 (0.78–1.55)	0.90 (0.63–1.28)	1.07 (0.67–1.71)	1.20 (0.78–1.85)	1.47 (1.01–2.12)	1.50 (0.96–2.34)	1.87 (1.26–2.78)
**Nutrition Facts label questions**								
Answered 1 question correctly	1.12 (0.66–1.88)	1.42 (0.99–2.03)	1.37 (0.93– 2.02)	1.18 (0.72 −1.95)	1.73 (1.11−2.70)	2.35 (1.60–3.45)	0.90 (0.58–1.41)	1.16 (0.78–1.77)
Answered both questions correctly	0.81 (0.44–1.49)	1.36 (0.90–2.06)	1.23 (0.78–1.93)	0.76 (0.41–1.43)	1.67 (1.02–2.74)	2.25 (1.44–3.53)	1.23 (0.73–2.07)	1.98 (1.23–3.20)

a Values are adjusted odds ratios (95% confidence intervals). Although a narrow CI suggests a more precise estimate, a wider CI should be interpreted with caution.

b Reference is “good.”

c Reference is “sometimes.”

d Reference is “never.”

e Reference is “somewhat harmful.”

## Discussion

Our study yielded 2 main findings. First, although participants appeared to understand the consequences of excess sodium intake, they did not know recommendations for daily sodium consumption or the sodium content of foods that are high contributors to salt in the American diet, as demonstrated by participants’ limited understanding of how sodium content is displayed on food labels. This finding supports previous work that suggests that the level of knowledge pertaining to daily sodium recommendations is low among LAC residents (6). Second, increased knowledge about the harmful effects of sodium was associated with increased engagement in some healthy behaviors, such as watching salt intake or declining a food purchase because of its salt content. This finding aligns with previous studies that found positive associations between increased knowledge of nutritional concepts and improved food choices (14,15). Although increased knowledge about specific sodium consumption recommendations was associated with increased use of Nutrition Facts labels to guide food purchasing decisions, this finding was conversely associated with lower odds of doing anything to control or prevent hypertension.

The LAC DPH continues to encourage residents to reduce salt consumption through an array of strategies, including applying nutrition standards to food venues such as hospitals and universities and modifying their menus. Results from our study suggest that LAC residents require further nutrition education to take advantage of increased availability of low sodium foods as a result of these implemented sodium reduction strategies. LAC DPH conducted the Salt Shocker campaign, including educational videos, to make residents aware of recommendations for sodium consumption and the amount of sodium in common foods that add significantly to the volume of salt in the American diet. For example, the campaign highlighted that 3 fast-food packets of ketchup (over 500 mg) and 1 cup of cottage cheese (900 mg) each contain over 20% of the *Dietary Guidelines for Americans*’ recommendation for daily sodium intake (13). While sliced bread and canned vegetables do not contain the highest amounts of sodium per serving of popular prepared foods, they contribute heavily to the amount of sodium Americans consume through their frequent use as ingredients in commonly prepared dishes. Consequently, CDC recommends that Americans choose low sodium or no-added-salt varieties of bread and canned vegetables (16). 

Findings from our study suggest that health education messaging, especially in regard to reducing sodium intake, should be integrated with policy and system-level change interventions such as those from recent chronic disease–related efforts (17). Previous studies found that residents of developed countries such as the United States and Canada are receptive to some, but not all, dietary sodium recommendations or warnings with differences in knowledge and receptiveness tied to socioeconomic status and race/ethnicity (14). Future campaigns should take into account that although recommendations and warnings about sodium intake are generally accepted (15), specific warnings against consumption of processed foods containing large amounts of sodium, such as breads or cereal, are rarely followed because most people are unable to correctly identify high-sodium foods (18,19). Furthermore, coupling these recommendations or this messaging to multifaceted nutritional interventions may be an effective way to raise public awareness about the dangers of excess salt consumption while simultaneously supporting the implementation of industry-focused efforts, including adherence to voluntary sodium limits for processed foods established by the US Food and Drug Administration (20–22). 

Industry acceptance of incremental reductions in the sodium content of processed foods, which are possible without affecting the taste or marketability of such foods, would allow for maximum effectiveness of nutrition education efforts by making low sodium foods more common (23,24). Increasing the market share of low-sodium foods, in addition to increasing knowledge about sodium and its potential health consequences, may improve health outcomes.

Our study has several limitations. First, time may have affected the responses of participants because the series of internet panel surveys was administered over a 2- to 3-year period. However, the sampling method used by Global Strategies Group attempted to make individual participants interchangeable across survey waves and to allow for an analysis of the data independent of time. Second, as with all cross-sectional designs, no causal relationships can be determined between predictors and outcomes; results from the logistic and multinomial regression models can only be interpreted as associations. Third, the nature of the Internet panel survey methodology is linked to potential selection bias, because participants may have self-selected because of the incentives given and because of their desire to contribute to this type of study. The final study population may also be skewed toward people with continuous Internet or computer access. Fourth, questions regarding the perceived sodium content of commonly consumed foods may have been interpreted with mixed accuracy. Although all the foods were promoted by prior health marketing campaigns as high contributors to dietary sodium, not all foods that were highlighted contained high amounts of sodium per single serving. Lastly, although many questions used in the Internet panel surveys were validated on the basis of their use in national-level surveys, questions about the Nutrition Facts label or the question about the food panel were designed by the DPH staff. These more tailored questions may or may not be valid when compared with similar questions used in similar studies.

Our study highlights the needs for local jurisdictions such as LAC to educate its residents about daily sodium recommendations. These results may inform the development and dissemination of future sodium reduction efforts and consumer messaging in LAC and elsewhere in the United States.
